# Pseudoglandular Schwannoma With *FUS::KLF17* Fusion: Broadening the Spectrum of *FUS*‐Associated Tumors

**DOI:** 10.1002/gcc.70077

**Published:** 2025-08-12

**Authors:** Jerome Givi, Daisy Wu, Rania Bakkar, Michelle Afkhami, Diana Bell

**Affiliations:** ^1^ Department of Pathology University of Pittsburgh Medical Center Pittsburgh Pennsylvania USA; ^2^ Department of Pathology City of Hope Cancer Center Duarte California USA

**Keywords:** *FUS*, *KLF17*, myoepithelial tumor, pseudoglandular schwannoma, schwannoma

## Abstract

We present a case of a 51‐year‐old male with a pseudoglandular cellular schwannoma arising from the brachial plexus, which contains the expected molecular aberrations for a schwannoma (chromosome 22q loss encompassing the *NF2* and *LZTR1* genes) as well as a *FUS*::*KLF17* rearrangement. Pseudoglandular schwannomas are rare morphologic variants of schwannomas that contain gland‐like spaces lined by S100‐positive, cytokeratin‐negative pseudocolumnar Schwann cells. Fusions involving *FUS* and *EWSR* are commonly found in myoepithelial tumors of bone and soft tissue. While the spectrum of tumors with fusions involving *FUS* and *EWSR* is relatively broad, no cases, to our knowledge, have been reported of schwannomas, let alone the morphologically distinct pseudoglandular schwannoma, containing a *FUS* rearrangement. This case thus expands the spectrum of *FUS* rearranged tumors, highlighting the need for documentation of similar cases to understand the clinical significance of this combination.

## 1 Case Presentation

A 51‐year‐old male was referred for a palpable left chest/axillary mass. MRI of the chest showed a 29 × 22 × 20 mm heterogeneously T2 hyperintense and predominantly peripherally enhancing mass in the lateral aspect of the pectoralis major muscle (Figure S1). The inferior component of this mass demonstrated more solid enhancement. The mass was excised in its entirety from the pectoralis major muscle and sent for permanent pathology review.

**FIGURE 1:**
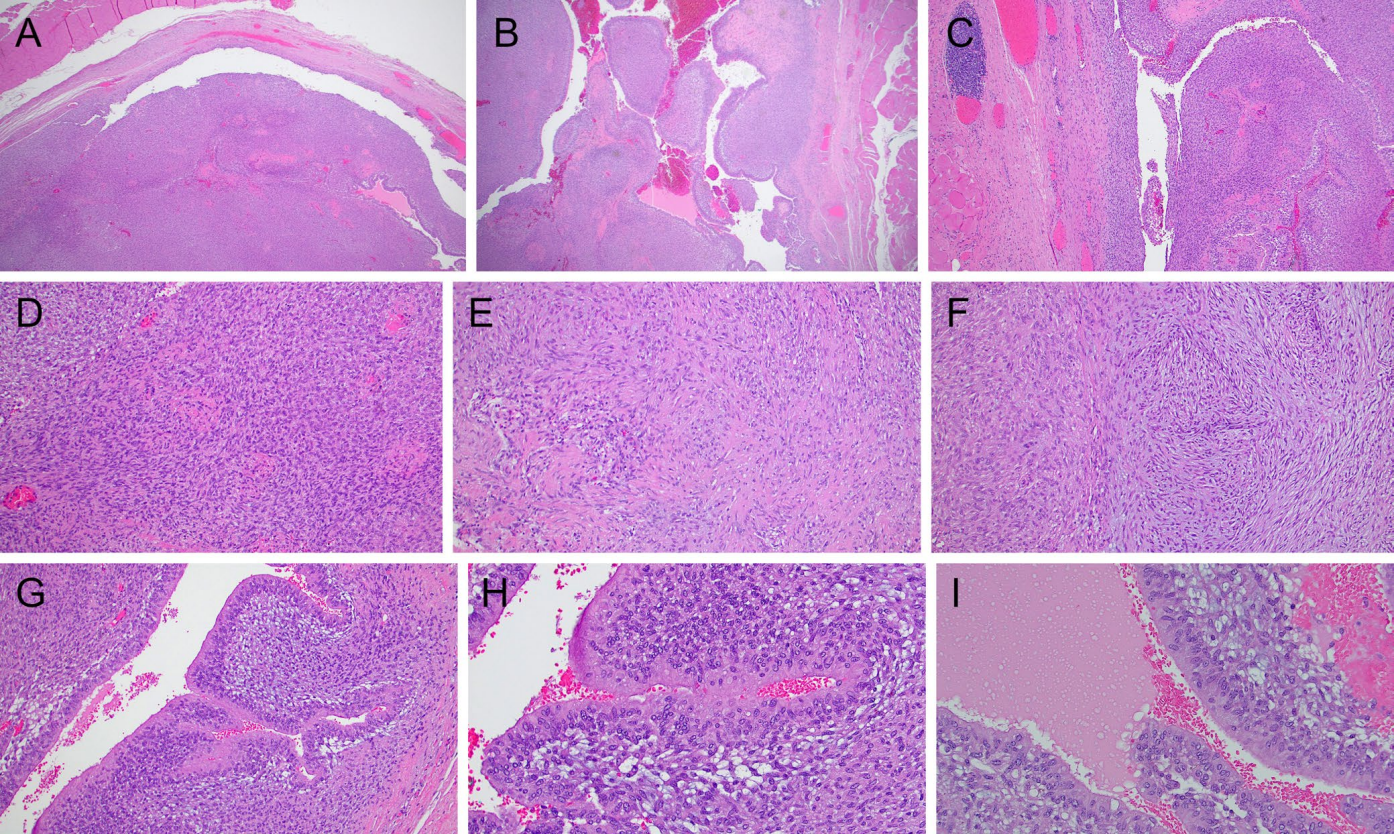
(A–I) Histological features (hematoxilin and eosin). A well‐circumscribed, solid cystic, biphasic spindle cell neoplasm (A, B, C); scattered lymphoid aggregates were present at the periphery of the neoplasm (C). Dominant morphology consisted of hypercellular regions of densely packed spindle cells (D); admixed less cellular regions of spindle cells (E, F). The lesion also contained cystic spaces and branching, gland‐like structures (E, F, G) lined by multilayered cuboidal cells (F) and occasionally filled by eosinophilic secretion‐like material (G).

**FIGURE 2:**
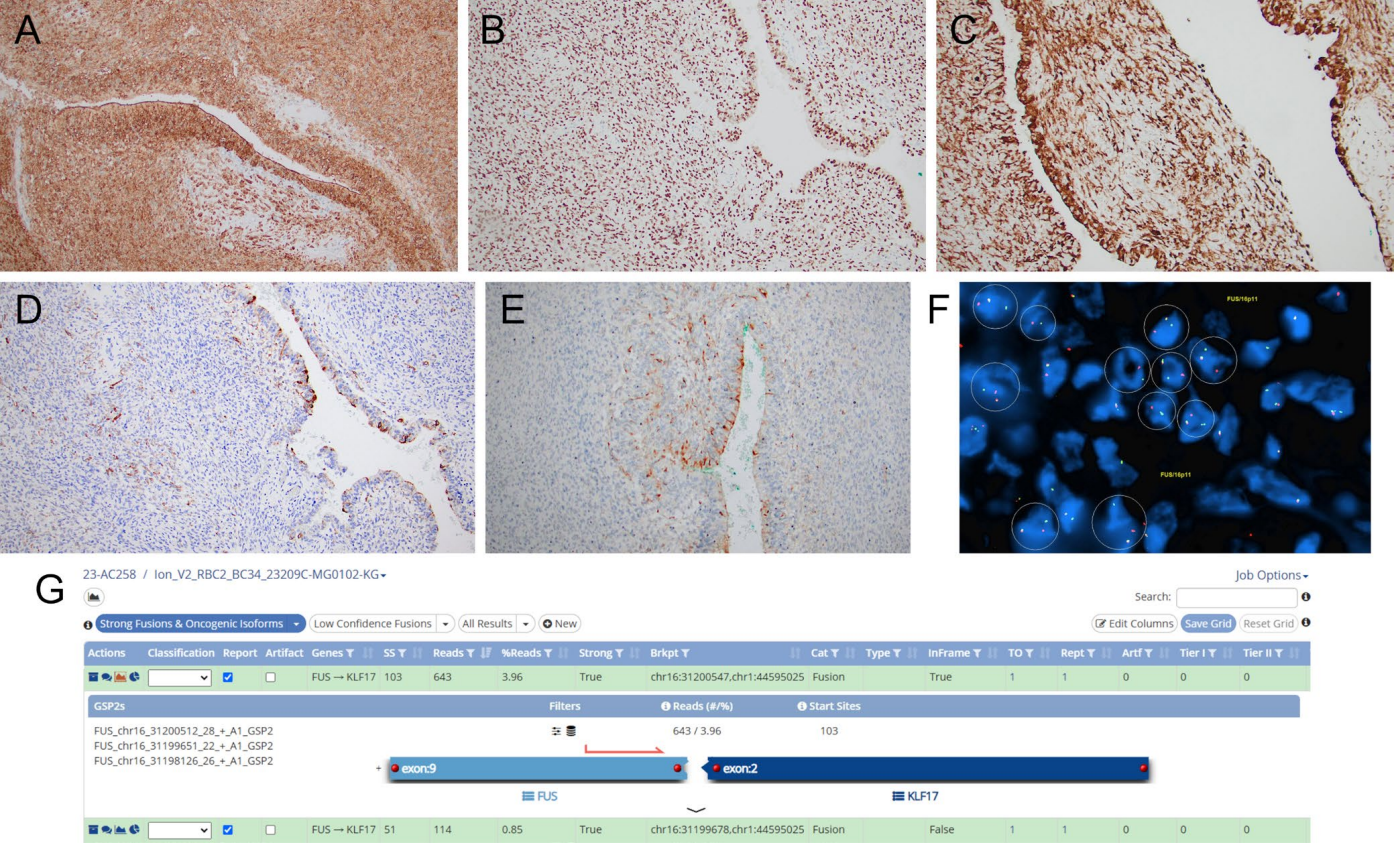
Phenotype characteristics (A–E). The stroma and pseudoglandular component are both strongly immunoreactive with anti‐S‐100 (A); anti‐Sox10 (B), anti‐p16 (C). Focal, scattered immunoreactivity with anti‐AE1/AE3 (D) and anti‐Cam5.2 (E) was present in the cells lining the pseudoglandular structures and stromal cells. Molecular findings (F, G). FUS rearrangement by FISH gives further credence to molecular RNASeq findings (F). Detection of *FUS::KLF17* fusion in pseudoglandular schwannoma—schematic illustration of the *FUS* (exon 9) gene to the *KLF17* (exon 2) gene in‐frame fusion (G).

Grossly, the resection specimen was a 4.0 cm irregular portion of muscle. Cross‐sectioning revealed a 2.4 × 1.3 × 1.2 cm well‐demarcated tan to yellow mass lesion. Microscopically, the mass was well‐circumscribed, solid‐cystic, biphasic, with dominant hypercellular regions of densely packed short fascicles of spindle cells; scattered and admixed, less cellular, focally myxoid regions of spindle cells; no well‐formed Verocay bodies (Figure 1). The lesion also contained cystic spaces and branching, gland‐like structures lined by multilayered cuboidal cells and occasionally filled by eosinophilic secretion‐like material (Figure 1). No necrosis, cytologic atypia, or pleomorphism was present, and mitoses were infrequent (2 per 2 mm^2^). Scattered lymphoid aggregates were present at the periphery of the neoplasm. Immunohistochemically, the spindled stroma and pseudoglandular component were both diffusely and strongly positive for S100, Sox10, and p16 (Figure 2). Cytokeratins AE1/AE3 and CAM5.2 were also focally weakly positive in the cells lining the pseudoglandular structures and stromal cells (Figure 2). No immunoreactivity was detected with CK7, p63, EMA, SMA, calponin, desmin, myogenin, CD34, Glut1; the proliferation rate was low (Ki‐67 focally up to 5%).

The differential diagnostic considerations included nerve sheath tumors (peripheral nerve sheath—schwannoma, or hybrid nerve sheath tumor) and myoepithelial tumor of soft tissue.

The case was sent for next generation sequencing using a 523 gene DNA panel to detect single nucleotide variants, copy number variants, microsatellite instability, and tumor mutational burden, and RNA sequencing of 165 genes. Molecular testing had several notable findings, including a chromosome 22q11.21–22q13.2 loss, including *NF2* and *LZTR1*, an *IGF2* gene amplification, and a *FUS::KLF17* fusion resulting from t(1;16) (Figure 2). The tumor was microsatellite stable and had an overall low tumor mutation burden. Fluorescence in situ hybridization (FISH) using a *FUS* dual break apart probe (16p11) was positive for translocation (Figure 2), providing further validation for the NGS reported finding. FISH using a dual color break apart probe for *EWSR1* (22q12) was negative for translocation.

Given the morphologic impression, immunoprofile, and molecular findings, a diagnosis of pseudoglandular cellular schwannoma, likely arising from the brachial plexus, was rendered. Twelve months following surgery, the patient showed no evidence of recurrence.

## 2 Discussion

Schwannomas are benign, slow‐growing tumors arising from Schwann cells that can occur sporadically or in association with neurofibromatosis type 2 (NF2) and schwannomatosis. They can occur throughout the body, but common sites of origin are the peripheral nerves in the skin and subcutaneous tissues of the head and neck, as well as the flexor surfaces of the limbs [1]. Schwannomas arise due to biallelic inactivation of the *NF2* gene on chromosome 22q12, which encodes merlin, a tumor suppressor protein. This biallelic inactivation can occur sporadically or be caused by a combination of germline and somatic *NF2* gene mutations in patients with NF2. In schwannomatosis (characterized by the presence of multiple schwannomas, generally occurring later and not involving the vestibular nerve), germline mutations in *SMARCB1* or *LZTR1* are common. The multistep tumorigenesis in these cases also involves *NF2* mutations [2]. Histologically, the tumors classically demonstrate a biphasic pattern of alternating Antoni A and B patterns, sometimes with Verocay bodies in Antoni A regions. Five schwannoma subtypes are defined in the WHO Classification of Tumors 5th: ancient, cellular, plexiform, epithelioid, and microcystic/reticular [1].

Pseudoglandular schwannomas contain gland‐like or cystic spaces lined by pseudocolumnar or cuboidal‐like neoplastic Schwann cells that can contain secretion‐like material and may have intraluminal and hemosiderin‐laden macrophages [3, 4, 5, 6, 7, 8, 9]. The cells lining these pseudoglandular spaces are positive for S100 and can be positive for GFAP but are consistently negative for cytokeratin (apart from one case report with slight focal positivity in cauda equina) [3, 4, 5, 7, 8, 10]. This is in contrast to true glandular elements that can sometimes be entrapped in schwannomas, which are negative for S100 and positive for EMA and cytokeratin [11]. Our case shows focal weak keratins AE1/AE3 and Cam5.2 immunoreactivity in the pseudoglandular spaces and stromal cells, while other epithelial markers (CK 7, EMA) were negative.

As only a few cases of schwannomas with pseudoglandular features were reported prior to 2000, this subtype was considered rare [3, 4]. However, a retrospective review of 202 schwannomas by Robinson et al. [5] revealed 16 (7.9%) schwannomas with pseudoglandular features. Ud Din et al. [6] performed a similar review of 971 schwannomas, reporting 61 (6.3%) cases with pseudoglandular elements, indicating that this feature may be more common than previously thought. Both authors favor the pseudoglandular spaces to be a result of cystic degeneration of Verocay bodies, but there have been no attempts at molecular characterization of schwannomas with pseudoglandular elements. Well‐formed, conventional Verocay bodies were not appreciated in our case.

Another diagnostic consideration was given towards a hybrid nerve sheath tumor (schwannoma with perineurioma and schannoma with neurofibroma), lack of EMA, Glut1, and CD34 immunoreactivity excluded perineurioma and neurofibroma components.

A tumor type commonly found to harbor fusions involving *EWSR* or *FUS*, including this *FUS::KLF17* fusion, is myoepithelial tumors (METs), a clinically and morphologically diverse group of neoplasms that can be found in the salivary gland, skin, or soft tissue. METs may contain ducts and range from benign to malignant, with nomenclature and features that define malignancy differing between sites. There is no specific immunostain for myoepithelial differentiation, but these tumors consistently co‐express epithelial markers such as cytokeratin and EMA, as well as S100. They may also express calponin, GFAP, SMA, p63, SOX10 [1]. In our case, other pertinent myoepithelial immunostains (SMA, calponin, p63) were negative; FISH for EWSR was also negative‐ making the diagnosis of MET less likely.

While morphology and immunophenotype overlap between METs in bone/soft tissue/viscera and skin/salivary glands, their genetics are quite different. Skin and salivary gland METs frequently contain rearrangements in the *PLAG1* and *HMGA2* genes [12, 13, 14]. Bone and soft tissue METs are most commonly found to harbor fusions involving *EWSR* or, less commonly, *FUS* genes [12, 13, 14]. Numerous fusion partners with *EWSR* have been described, including *POU5F1, PBX1, PBX3, ZNF444, KLF15, KLF17*, and *ATF1* [13, 14]. The most common fusion partner of *FUS* is *KLF17*; although cases with *FUS*::*POU5F1* have also been described [13, 14, 15].

*FUS* and *EWSR* are members of the TET family and code for nuclear RNA/DNA‐binding proteins primarily involved in RNA metabolism, DNA repair, and cellular processes like transcription, splicing, and mRNA transport. These genes can substitute for each other as translocation partners in METs and various other neoplasms, including myxoid liposarcoma (*FUS*/*EWSR*::*DDIT3*), low‐grade fibromyxoid sarcoma (*FUS*/*EWSR*::*CREB3L1* or *CREB3L2*), angiomatoid fibrous histiocytoma (*FUS*/*EWSR*‐*ATF1*), and Ewing sarcoma, among other sarcomas and some hematologic malignancies [15]. The most common fusion partner of *FUS* in METs is *KLF17*, a transcriptional regulator that empowers the TGF‐β/SMAD signaling pathway, which typically suppresses tumor growth and metastasis [15]. The translocation breakpoint occurs in the *KLF17* 5′‐untranslated region, so it is thought to disrupt the translation or function of *KLF17* [15].

To our knowledge, the spectrum of *FUS*/*EWSR*‐rearranged tumors has, until now, not included schwannomas. This case is exceptional because it presents a cellular schwannoma with pseudoglandular features, a rare morphologic variant, containing a *FUS*::*KLF17* fusion along with a 22q loss that is common among schwannomas. This combination of histological features and molecular findings is previously unreported, expanding the spectrum of *FUS*‐associated tumors and underscoring the importance of molecular testing in diagnosing and understanding rare tumor types.

## 3 Conclusion

We present a highly unusual case of a cellular schwannoma with pseudoglandular features exhibiting an expected chromosome 22q loss encompassing the *NF2* and *LZTR1* genes, as well as a *FUS*‐*KLF17* fusion. The presence of pseudoglandular elements, characterized by S100 positive, gland‐like structures lined by Schwann cells, represents a rare morphological variant of schwannoma. The co‐occurrence of a *FUS*‐*KLF17* fusion is notable, as this genetic abnormality has primarily been reported in myoepithelial tumors and never in schwannomas. This combination is, to the best of our knowledge, previously unreported; thus, expanding the spectrum of *FUS*‐associated tumors. Further research and the documentation of similar cases are essential to understand the clinical significance of this combination.

## Author Contributions

Jerome Givi, Daisy Wu, Rania Bakkar, Michelle Afkhami, and Diana Bell contributed to the conception and design of the work, acquisition, and interpretation of data, drafting the MS, and revising it. All the authors have read and approved the final manuscript.

## Ethics Statement

In accordance with institutional ethical guidelines.

## Conflicts of Interest

The authors declare no conflicts of interest.

## Supporting information


**Figure S1:** Imaging of chest (MRI and ultrasound) showed a 2.9 cm heterogeneously T2 hyperintense and peripherally enhancing mass in the lateral aspect of the left pectoralis major muscle (MRI, T1 sequence—left; MRI T2 sequence—right upper; ultrasound—right lower).

## Data Availability

The data that support the findings of this study are available on request from the corresponding author. The data are not publicly available due to privacy or ethical restrictions.
