# Supplementary figures and images for: Pseudoglandular Schwannoma With FUS::KLF17 Fusion: Broadening the Spectrum of FUS‐Associated Tumors
Source: Genes Chromosomes Cancer. 2025 Aug 12;64(8):e70077. doi: 10.1002/gcc.70077 (PMC12340572; doi:10.1002/gcc.70077)

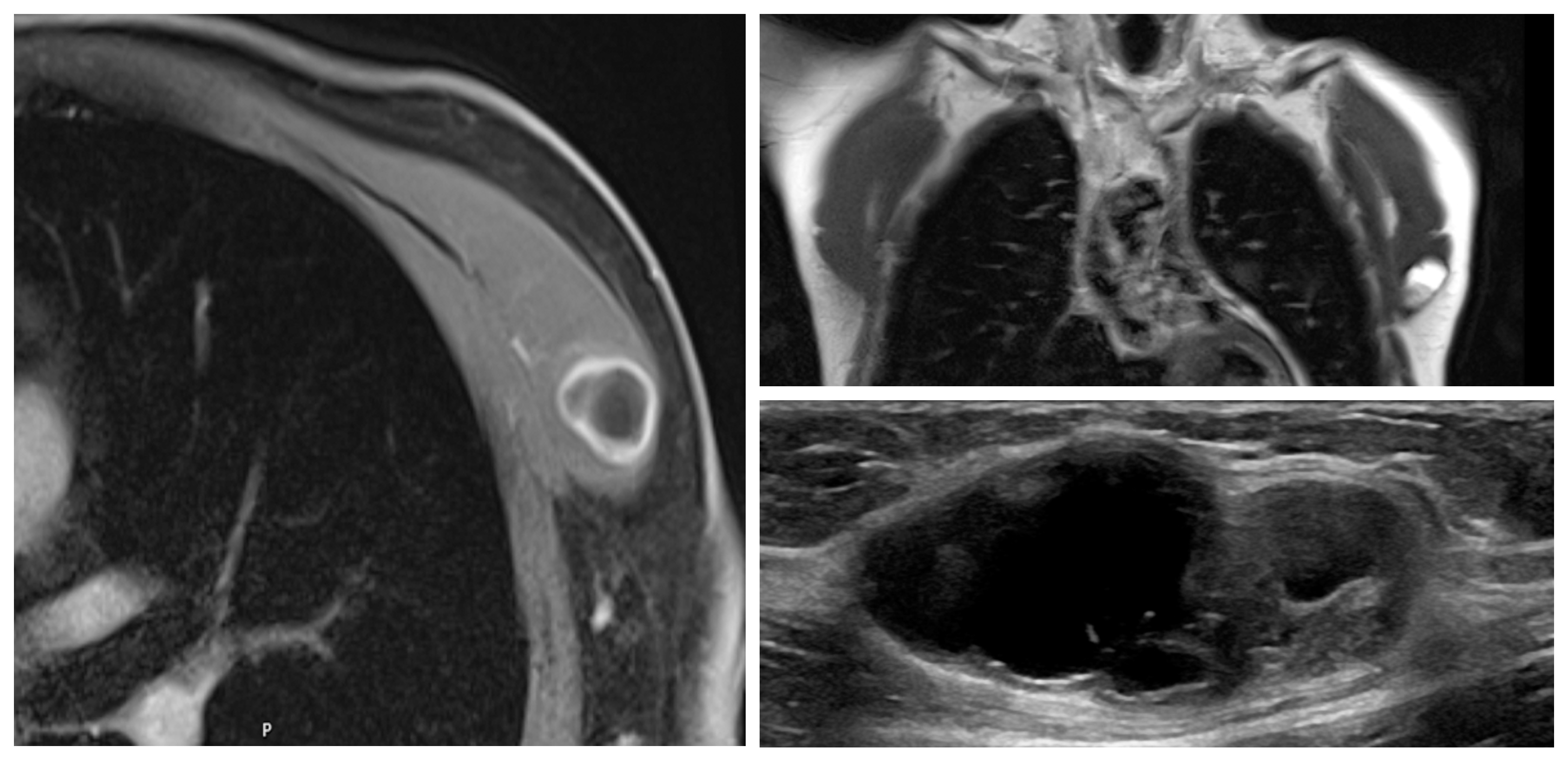

Supplement: Supplementary file 1 — Figure S1: Imaging of chest (MRI and ultrasound) showed a 2.9 cm heterogeneously T2 hyperintense and peripherally enhancing mass in the lateral aspect of the left pectoralis major muscle (MRI, T1 sequence—left; MRI T2 sequence—right upper; ultrasound—right lower). [file GCC-64-e70077-s001.jpg]
